# Beta-Lapachone, a Modulator of NAD Metabolism, Prevents Health Declines in Aged Mice

**DOI:** 10.1371/journal.pone.0047122

**Published:** 2012-10-11

**Authors:** Jeong-sook Lee, Ah Hyung Park, Sang-Hee Lee, Seoung-Hoon Lee, Jin-Hwan Kim, Suk-Jin Yang, Young Il Yeom, Tae Hwan Kwak, Dongyeop Lee, Seung-Jae Lee, Chul-Ho Lee, Jin Man Kim, Daesoo Kim

**Affiliations:** 1 Department of Biological Sciences, KAIST (Korea Advanced Institute of Science and Technology), Daejeon, Korea; 2 Department of Pathology and Infection Signaling Network Research Center, Chungnam National University School of Medicine, Daejeon, Korea; 3 R&D Institute, Mazence Inc., Suwon, Korea; 4 Medical Genomics Research Center, KRIBB (Korea Research Institute of Bioscience and Biotechnology), Daejeon, Korea; 5 Division of Molecular and Life Science, POSTECH (Pohang University of Science and Technology), Pohang, Kyungbuk, Korea; 6 Animal Model Center, KRIBB (Korea Research Institute of Bioscience and Biotechnology), Daejeon, Korea; University of Texas Health Science Center at San Antonio, United States of America

## Abstract

NADH-quinone oxidoreductase 1 (NQO1) modulates cellular NAD^+^/NADH ratio which has been associated with the aging and anti-aging mechanisms of calorie restriction (CR). Here, we demonstrate that the facilitation of NQO1 activity by feeding β-lapachone (βL), an exogenous NQO1 co-substrate, prevented age-dependent decline of motor and cognitive function in aged mice. βL-fed mice did not alter their food-intake or locomotor activity but did increase their energy expenditure as measured by oxygen consumption and heat generation. Mitochondrial structure and numbers were disorganized and decreased in the muscles of control diet group but those defects were less severe in βL-fed aged mice. Furthermore, for a subset of genes associated with energy metabolism, mice fed the βL-diet showed similar changes in gene expression to the CR group (fed 70% of the control diet). These results support the potentiation of NQO1 activity by a βL diet and could be an option for preventing age-related decline of muscle and brain functions.

## Introduction

It has been recently suggested that NQO1 (NAD(P)H:quinone oxidoreductase) is involved in the mechanism of aging; NQO1 modulates the cellular NAD^+^/NADH ratio associated with aging and age-related disorders [1] by reducing substrates using NADH as an electron donor [2], [3]. The enzymatic function of NQO1 is decreased in aged tissues and enhanced by calorie restriction (CR), a beneficial strategy for body health and life-span [4]. Consistently, the overexpression of NQR1, a yeast homolog of mammalian NQO1, increases the lifespan of yeast [5]. It remains unclear yet if the direct modulation of NQO1 activity is also beneficial in mammalian species. A lack of small molecules that stimulate NQO1 activity has been one of the major hurdles to address the issue.

The quinone-containing compound, β-lapachone (3,4-dihydro-2,2-dimetyl-2H-naphthol[1,2-b]pyran-5,6-dione; [βL]), originally obtained from the Lapacho tree, has been used for medical purposes [6], [7]. Both *in vitro* and *in vivo*, βL facilitates the NQO1-dependent oxidation of NADH to NAD^+^ by receiving two electrons from NADH [3]. The capacity of βL to modulate NAD turnover is totally dependent on NQO1 as these effects were abolished in NQO1 knockout cells [8]. Inspired by the fact that CR also modulates the cellular NAD^+^/NADH ratio in aged tissues [9], [10], here we compare the effect of βL on behavioral, biochemical, and molecular parameters of aging in aged mice with those of CR and describe the effectiveness of βL-supplemented diet in the prevention of aging related mechanisms.

## Materials and Methods

### Ethics Statement

All procedures used in animal experiments were performed according to a protocol approved by the Animal care and Use committee of Korea Advanced Institute of Science and Technology (protocol number KA2010-12, KA2012-04).

### Animals and diets

Male C57BL/6 mice at 6 months of age were purchased (the Orient Bio, Korea) and maintained on a standard chow (11.9 kcal% fat ; Labdiet, 5053PicoLab Rodent Diet20) prior to the start of the experiment. Thirteen-month-old mice were maintained on three different diets: a normal diet in the control group (C group, *Ad libitum*), calorie restriction (CR group, 70% of normal diet), or a normal diet with the addition of 0.066% β-lapachone (βL group). Average daily doses of βL over the course of the experiment were approximately 70∼80 mg/kg/day which is calculated by (food intake (g) * 0.66 mg/g)/(body weight(g)/1000 g/kg). The mice were maintained on a 12 hour light/dark cycle and with 22–24°C. Body weight and food intake were measured on a weekly basis throughout the experiments.

### Measurement of NAD+/NADH levels

Frozen tissue samples were extracted by 1M perchloric acid (HClO_4_) or 1M potassium hydroxide (KOH) solution to determine oxidized and reduced pyridine nucleotide contents. Extracted samples were subjected to ultrasonication with a sonic Dismembator (Fisher Scientific, Fairland, NJ). After centrifugation at 12,000 rpm for 10 min, the supernatant was filtered through a Microcon YM-3 filter (Millipore, Bedford, MA). Electrospray-ionization mass spectrometry was performed in negative ion mode using MDS Sciex API 4000 Triple Quadrupole Mass Spectrometer (Applied Biosystems, Ontario, CA) followed by chromatographic separation on an Agilent 1100 series HPLC system (Agilent technologies, Palo Alto, CA) equipped with a XTerra MS C18 2.1×150 mm, 3.5 µm column (Waters, Milford, Massachusetts, USA).

### Behavioral analysis

#### Rotarod test

The mice were placed on a rotarod device (RotaRod 7650, Ugo Basile, Comerio, Italy) and the latency for the mice to fall off from an accelerating rotarod (2 to 40 rpm over 5 min) were measured. Each mouse was given four trials and a 30 min resting period between each trial.

#### Grip test

After the grip strength meter was placed horizontally, mice were allowed to grasp the triangular metal pull bar (with forelimbs only) and were then pulled backward in the horizontal plane. The force applied to the bar at the moment that the grasp was released was recorded as the peak tension (N). The test was repeated five times consecutively and the highest value was recorded.

#### Pole test

A mouse was place on the vertical bar (1 cm in diameter and 50 cm long) with headside-up, and the time taken for the mice to orient its body upside-down was measured. If the mouse slid down the pole or remained hanging for two minutes, the mouse failed that trial. Each mouse was given five trials.

#### Fear conditioning

Fear conditioning was conducted in a conditioning chamber (clear Plexiglas, dim light, metal grid floor; San Diego Instruments, USA) and a testing chamber (white plastic, circular cylinder) with video cameras mounted on the top of the chamber. The experiment was conducted over two days. On day one, each mouse was acclimated to the conditioning chamber (4 min 40 s) and then given three pairings of a conditional stimulus (tone, 20 sec, 5 kHz, 75 dB) that co-terminated with an unconditional stimulus (foot shock, 2 sec, 0.7 mA). The trial interval was 60 sec. On the day of testing, the freezing responses to the conditional stimulus were measured in the testing chamber with test tones (180 sec). To test contextual conditioning, the mice were placed in the conditioning chamber and were allowed to explore for five minutes. An observer who was blinded to the previous treatments of the mice measured freezing manually.

### Analysis of physiological indicators

For indirect calorimetry, individual mice were placed in a chamber (Accuscan instruments, USA). Energy expenditure was calculated by measuring O_2_ consumption and CO_2_ production every 10 min for 12 h. At the same time, physical activity was measured. Blood samples were collected in heparinized tubes and separated by centrifugation. Enzyme-linked immunosorbent assay (ELISA) was used to quantify blood plasma insulin, adiponectin and leptin (Linco Research, MO). Enzymatic assays were used to quantify triglycerides, cholesterol and glucose, LDL and HDL (Beckman Instruments, CA).

### Electron microscopy

Mice were perfused under deep anaesthesia by the transcardial perfusion of heparinized saline followed by a fixative containing 4% paraformaldehyde and 0.1% glutaraldehyde in 0.1 M phosphate buffer. The muscles were removed and post-fixed overnight in the same fixative. Then the tissue was dissected out and cut perpendicular to the longitudinal axis for transmission electron microscopy (Tecnai G2 Spirit Twin; FEI Company; Korea Basic Science Institute).

### Quantitative-PCR (qPCR)

Three micrograms of the total RNA was used for cDNA synthesis with Superscript II (Invitrogen). Diluted cDNA samples were subjected to qPCR with specific primers for individual genes and a SYBR Green dye (Invitrogen) using a real-time PCR system (iQ5; Bio-Rad). The results were normalized to cyclophilin A. The forward and reverse primers used for each gene were as follows:

SIRT1 F: 5′-AACTTCACAGCATCTTCAAT-3′ R: 5′-TGACACTGTGGCAGATTGTTATT-3′; SIRT3 F:5′-CACTACAGGCCCAATGTCAC-3′ R: 5′-TCACAACGCCAGTACAGACA-3′; PGC1α F:5′-TTTCATT CGACCTGCGTAAA-3′ R:5′-GGAATGCACCGTAAATCTGC-3′; NRF1 F: 5′-TGATGGAGAGGTCCAACAAA-3′ R: 5′-GGTTTCCCCAGACAGGACTA-3′; mTFA F: 5′-ATACCTTCGATTTTCCACAGAAC-3′ R: 5′-ATACCTTCGATTTTCCACAGA AC-3′; UCP1 F: 5′-GGGACCTACAATGCTTACAGAGT-3′ R:5′-GTACAATCCACTGTCT GTCTGGA-3′; UCP3 F: 5′-TTCTACACCCCCAAAGGAAC-3′ R:5′- AATCGGACCTTCAC CACATC-3′; Cyclophilin A F:5′-TTTGCAATCCTGCTAGACTTGA-3′ R:5′- CCCCATCTG CTCGCAATA-3′.

### Microarrays

Total RNA was extracted using the RNeasy Mini Kit (QIAGEN), and the quality of the total RNA samples was assessed using an Experion automated electrophoresis system (Bio-Rad Laboratories). Then, 0.5 µg of total RNA from each sample was labeled using the Illumina TotalPrep RNA Amplification Kit (Ambion) in a process involving cDNA synthesis and *in vitro* transcription. Single-strand RNA (cRNA) was generated and labeled by incorporating biotin-NTP (Ambion). A total of 1.5 µg of biotin-labeled cRNA was hybridized to a Sentrix mouse-8 v2 Expression BeadChip (Illumina) for 16 hours at 58°C. The hybridized biotinylated cRNA was detected with streptavidin-Cy3 and quantitated using Illumina's Bead Array Reader Scanner (Illumina). Array data were processed and analyzed by BeadStudio version 3.0 software (Illumina). Data normalization was performed using quantile normalization and fold changes and statistical significance were determined using the Avadis Prophetic version 3.3 (Strand Genomics).

### Immunoblotting

Total proteins from tissues were extracted in RIPA lysis buffer (500 mmol/l Tris-HCl pH 7.4, 1 mmol/l EDTA, 150 mmol/l NaCl, 1% NP-40, 0.25% Na-deoxycholate, and 1 mmol/l phenylmethylsulfonl fluoride) and content was determined using the Bio-Rad dye binding microassay. Protein was electrophoresed on a SDS-polyacrylamide gel after boiling for 10 min with SDS sample buffer. Anti-AMPKα Ab and Anti-phospho-T172 AMPKα Ab were purchased from Cell Signaling Technology (Beverly, MA).

### Statistical analysis

All data were compared with Student's *t*-test (or paired t-test) for paired groups or repeated measure analysis of variance (one-way or two-way) using the SigmaStat 3.1™ software package (Jandel Scientific Software, Germany), followed by analysis using the post-hoc Holm–Sidak test. The data in this study are presented as the mean ± SEM. P<0.05 was considered statistically significant. The Kaplan-Meier method was used to estimate survival and survival rates were analyzed by the log-rank test.

## Results

### βL increases the NAD+/NADH ratio and the activation of AMPK pathways in aged mice

**Figure 1:**
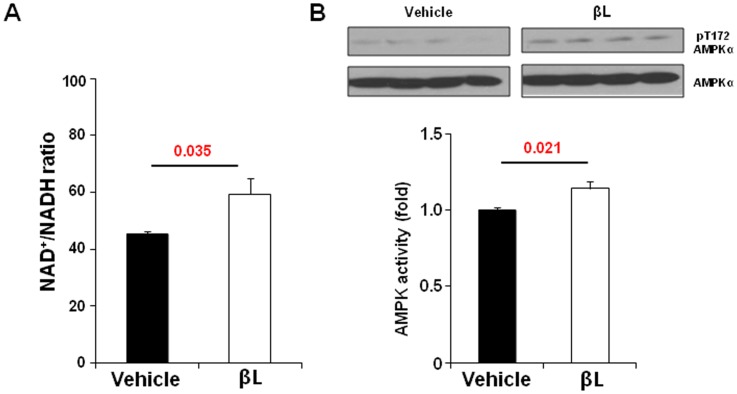
βL activates AMPK signalling pathway. (A) βL increases NAD^+^/NADH ratio by acute injection. NAD^+^/NADH ratio was calculated in the tissue of mice intravenously with vehicle (n = 5) or 5 mg/kg βL (n = 5) (B) Effect of βL on phosphorylation of AMPK for EDL (n = 5 per each group). Values represent mean± SEM.

We tried to examine the effect of βL on NAD+/NADH ratio in aged mice. When βL was injected (5 mg/kg, *i.v.* injection), the NAD^+^/NADH ratio was significantly increased in the muscle of aged mice (Fig. 1A). Consistently, in the extensor digitorum longus (EDL) muscle of aged mice, phosphorylated AMPK which is known to be enhanced by NAD^+^-dependent signaling pathways [11] was increased in response to βL treatment (Fig. 1B). These results suggest that the amount and the level of NQO1 activity in aged tissues is enough for boosting the βL-mediated production of NAD^+^ and NAD^+^-associated signaling pathways *in vivo*.

### Feeding βL mimicked CR effects on body weights and fats in aged mice

To compare the effects of βL with those of CR, cohorts of middle-aged (13-month-old) male C57BL/6J mice were provided with different dietary conditions: control diet (C, normal diet *ad libitum*), calorie restriction (CR, 70% of control diet), and β-lapachone supplemented food (βL, normal diet with the addition of 0.066% β-lapachone).

**Figure 2:**
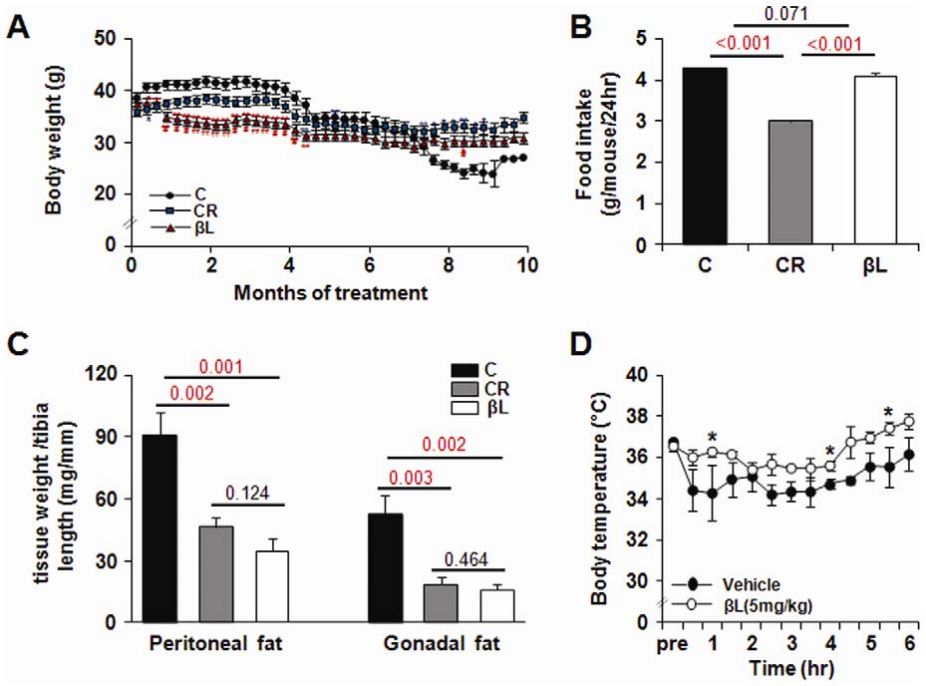
βL decreases body mass. (A) Body weight was measured over 10 months for all survivors. (B) Food intake was measured and calculated as average daily values for all survivors. (C) Fat mass was measured after 10months treatment (n = 5) (D) Changes in body temperature after injection with vehicle (n = 4) or 5 mg/kg βL (n = 4). Values represent mean±SEM. *p<0.05 **p<0.001 versus control ^#^p<0.05 ^##^p<0.001 versus CR.

Within 2–3 months, the βL-supplemented diet led to a decrease in body weight compared to the level found in young adult mice (Fig. 2A). During the total treatment period, the βL group showed a similar amount of food intake compared with the control group (Fig. 2B). The weights of peripheral and gonadal fats in βL-diet mice were significantly decreased (Fig. 2C). In addition, aging-related markers in blood, such as increased glucose, cholesterol and leptin associated with increased body fats [12] were significantly lowered in the βL and CR groups (Table 1).

**Table 1:** Effects on metabolic parameters in plasma.

Parameter	Control	Calorie restriction	β lapachone
GOT	149±11.8	156.9±17.1	173.7±26.7
GPT	46.1±4.0	64.2±3.9*	53.6±6.5
Cholesterol	121.7±11.4	87.5±7.0*	93.0±4.5*
Triglycerides	101.6±32.6	30.4±4.8*	84.5±16.7#
HDL	64.7±6.3	55.1±4.3	55.7±2.5
LDL	8.1±0.8	6.1±0.7	7.9±0.7
LDH	807.9±59.5	767.4±43.3	1013.0±109.2
Creatinine	0.2±0.02	0.2±0.01	0.2±0.01
IP	7.6±0.8	7.0±0.6	7.8±0.4
BUN	28.1±1.4	26.7±1.6	28.0±0.8
Glucose	214.0±39.9	184.0±9.6	166.0±9.6
Insulin	565.7±242.3	435.6±82.8	285.7±47.3
Leptin	9.6±3.5	8.6±1.3	2.9±0.6* #
Resistin	1.5±0.1	1.0±0.1*	1.3±0.1#

Data are the mean±s.e.m. (n = 5–10 per group).

* p<0.05 versus control;

# p<0.05 versus Calorie restriction.

How does βL lead to weight loss under normal food intake? We measured body temperature after injecting vehicle or βL (5 mg/kg, *i. v*) and found that βL maintained body temperature higher than vehicle control (Fig. 2D), suggesting that βL may lead to a higher energy expenditure resulting in weight loss and more evaporation heat energy.

### Feeding βL increases body metabolism

**Figure 3.βL:**
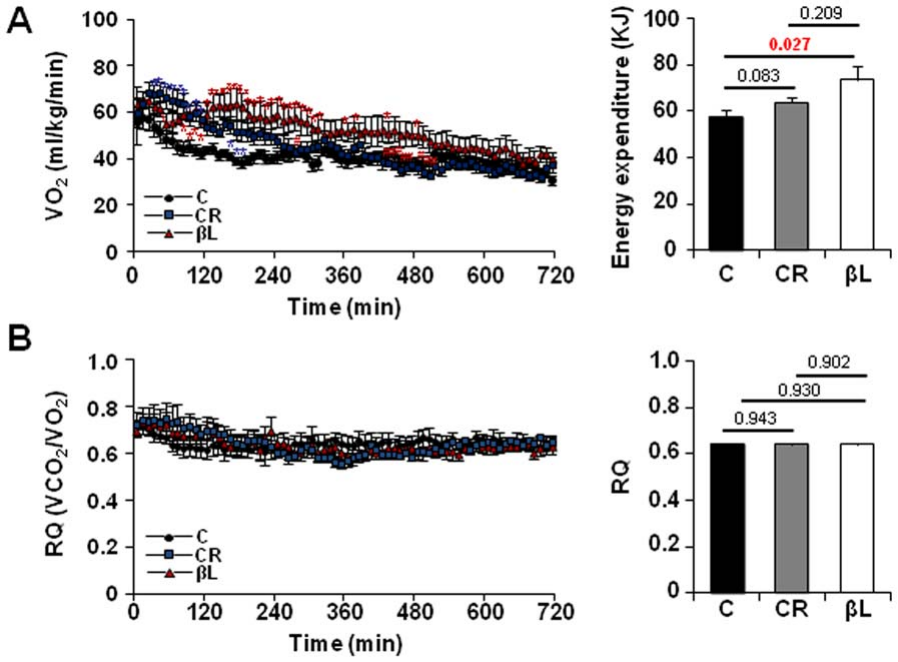
βL increases energy expenditure. (A) Energy expenditure as measured by changes in VO_2_ consumption in indirect calorimetry over 12 hr (n = 7 for each group). (B) Respiratory quotient (RQ) was calculated from the ratio of VCO_2_/VO_2_. Data represent the mean ± SEM. *p<0.05 **p<0.001 versus control ^#^p<0.05 ^##^p<0.001 versus CR.

**Figure 4:**
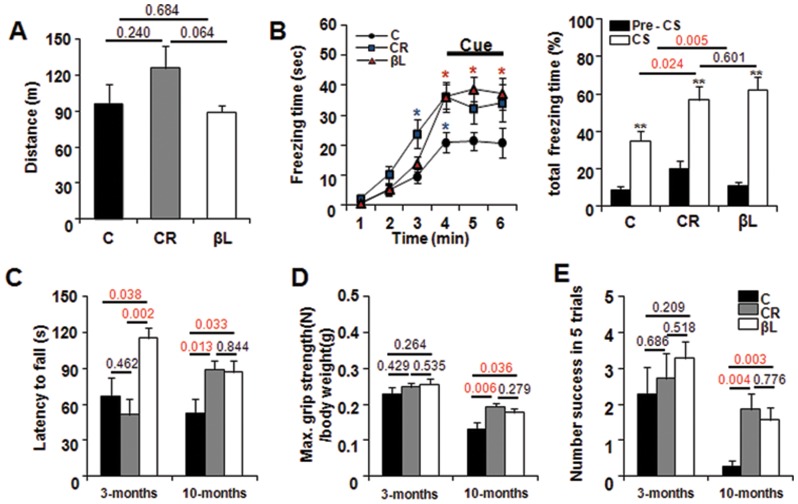
βL delays functional decline. (A)Total distance (B) Measurement of freezing time for cued conditioning (C)Time to fall from an accelerating rotarod (D)Peak tension measurements of grip strength (E)Number of successful trials in pole tests (n = 10–15 for each group) for control (C), calorie restriction (CR), and β-lapachone supplemented (βL) mice. Data represent the mean ± SEM. *P<0.05 versus control; **P<0.001 versus pre-CS.

To examine the degree of energy expenditure (EE) which contributes to energy homeostasis [13], EE values among the three groups were compared by measuring the oxygen consumption. Mice fed on a βL-supplemented diet showed a higher EE value compared to the control group (Fig. 3A) without an increase in locomotor activity (Fig. 4A). The respiratory quotient (RQ) values (VCO_2_/VO_2_), which depend on the type of fuel used (*i.e.*, glucose or lipids), showed no difference among the three groups (Fig. 3B). These results suggest that βL boosts the basal metabolic rate of aged mice, which is consistent with the fact that the metabolic rate can be modulated by NAD-associated signaling pathways [11].

### Feeding βL improves behavioral functions

To examine the functional aspects of the anti-aging effects of βL, mice were analyzed employing various behavioral paradigms. Both the CR and βL-fed groups had a higher capacity of associative memory than the control group, as measured by fear conditioning (Fig. 4B), although the three groups showed no significant differences in an elevated plus maze (EPM) test, which measures anxiety in mice (data not shown). In addition, both the CR and βL-fed groups showed higher motor performance and muscular strength than the control group (Fig. 4C–E). The effects of βL on motor functions appeared earlier than the CR effects (Fig. 4C–E). These data clearly indicate that βL mimics the effects of CR to ameliorate age-dependent decreases in body function [14], [15].

### Feeding βL prevents mitochondrial degenerations

**Figure 5:**
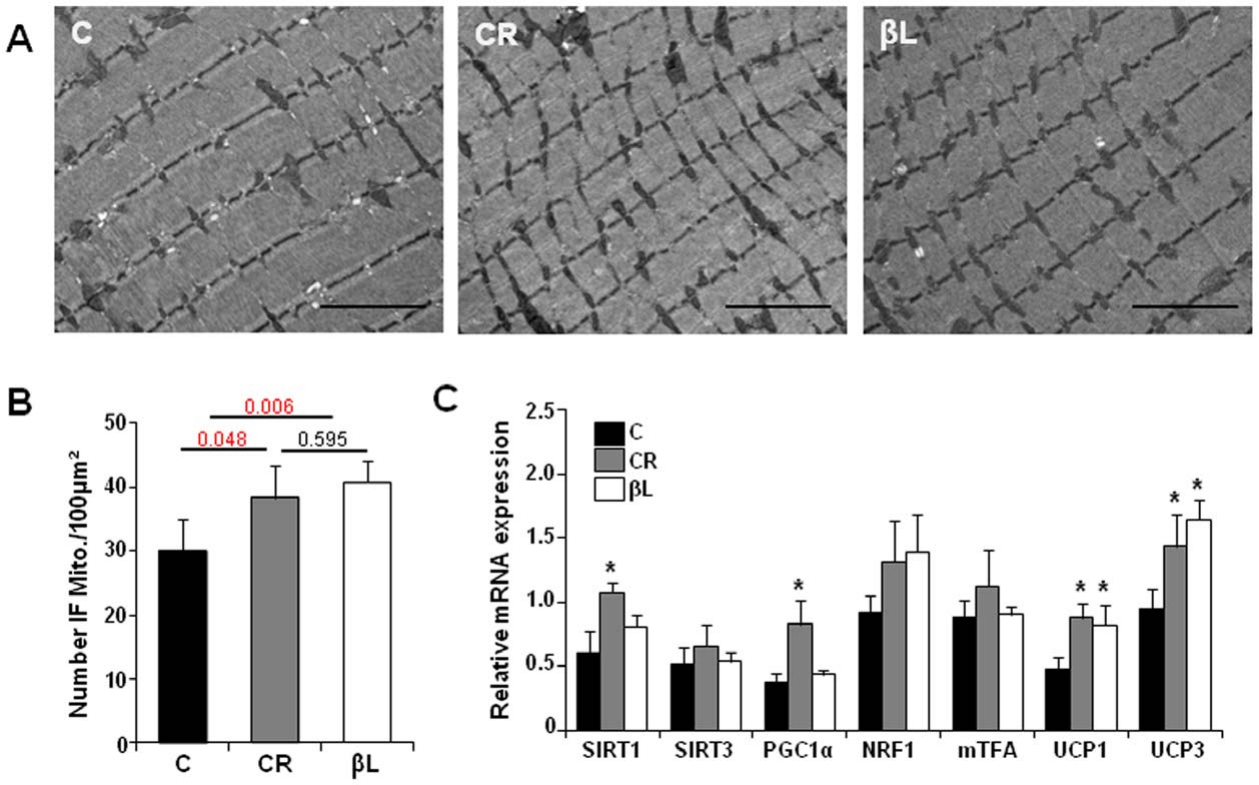
βL preserves mitochondria. (A) Electron microscopy (×15,000 magnification) of skeletal muscle. Scale bar represents 2 µm. (B) Quantification of inter-myofibrillar mitochondria number per 100 µm^2^ in the skeletal muscles after 10 months treatment (analysis of seven images per mouse, n = 5 per group) (C) Quantitative RT-PCR of genes related to mitochondrial function (n = 4 mice/group). Data represent mRNA levels relative to cyclophilin A. Data represent the mean ± SEM. *P<0.05 versus control.

Since mitochondrial function has been implicated in energy homeostasis in physiological and pathological states, we examined subcellular structures using electron microscopy. In aged mice with a normal diet, a substantial number of mitochondria were missing that are normally located perpendicular to the Z disks with regular spacing. In addition, some of them had abnormal mitochondrial cristae structures and elongated morphology (Fig. 5A). In contrast, the skeletal muscles of the CR and βL-diet groups had a higher number of intact mitochondria with regular spacing along the Z disks (Fig. 5B); both βL-diet and CR groups had more mitochondria with normal structure than the normal diet group, which is probably due to the resistance to mitochondrial degeneration or fusion known to be seen in aging [16].

Then, quantitative PCR analysis was performed to examine mitochondrial proteins that are known to reduce the production of ROS, which is a major cause of damage to cellular organelles. Two uncoupling proteins, UCP1 and UCP3, were significantly increased in CR and βL-diets groups when compared with controls (Fig. 5C). These results suggest that βL-diet increases metabolic rates and prevents mitochondrial damage, at least in part, by increasing UCPs [17].

### CR and βL diets induce similar gene expression changes

**Figure 6:**
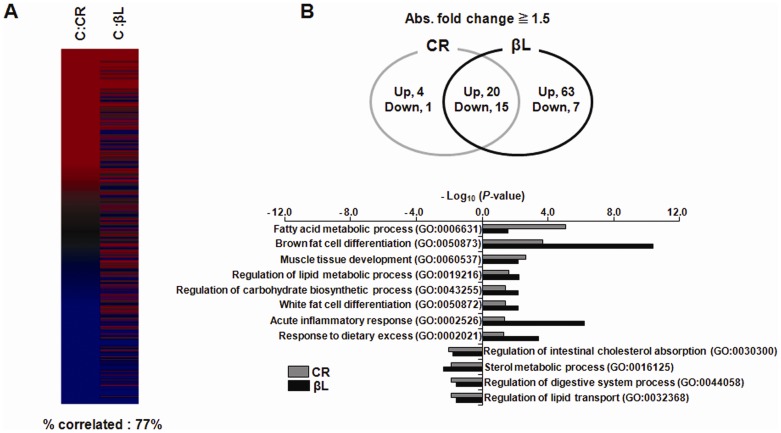
βL shifts expression patterns towards those for calorie restriction. (A) Parametric analysis of gene-set enrichment (PAGE) (up-regulated (red) or down-regulated (blue)) (B)(upper) Venn diagram shows number of overlapping genes between data sets.(lower) Gene ontology analysis. Results are plotted as negative log-transformed *p*- values.

To compare the molecular effect of βL diet on muscles with CR, we analyzed gene expression profile using GO gene sets and parametric analysis of gene set enrichment (PAGE) and found that both group showed a 77% correlation (Fig. 6A). In addition, aging-related genes [18], [19] were down- or up-regulated with similar patterns (Fig. 6A–B). Genes associated with muscle development and differentiations were up-regulated whereas genes involved in lipid transport were down-regulated (Fig. 6C, Table 2); for example, Csrp3 (cysteine and glycine-rich protein 3), one of the genes increased in βL group, is known to enhance skelectal myogenesis [20] and serine prepetidase inhibitor 1b and 1d (Serpina 1b and 1d) which are down-regulated and known to prevent obesity and insulin resistance [21]. These results are consistent with the βL effects on behavior (Fig. 4) and muscular structures and also explain how a βL- diet prevents aging-related changes.

**Table 2:** The most highly up-regulated and down-regulated genes in skeletal muscle.

Gene	Fold Change(CR/C)	Fold Change(βL/C)	Accession number	Definition
Genes up-regulated				
Myl2	7.20	13.98	NM_010861	Myosin, light polypeptide 2
Myl3	4.51	8.27	NM_010859	Myosin, light polypeptide 3
Sln	4.04	4.36	NM_025540	Sarcolipin
Scd1	3.27	2.32	NM_009127	Stearoyl-coenzyme A desaturase 1
Tnnc1	3.04	10.00	NM_009393	Troponin C
Csrp3	2.88	9.68	NM_013808	Cysteine and glycine-rich protein 3
Per1	2.47	2.97	NM_011065	Period homolog 1
Dbp	2.44	2.39	NM_016974	D site albumin promoter binding protein 3
Tnnt1	2.42	8.52	NM_011618	Troponin T1
Per2	2.35	2.20	NM_011066	Period homolog 2
Cyp2e1	2.31	2.25	NM_021282	Cytochrome P450, family2, subfamily e, polypeptide 1
Myh2	2.26	2.61	NM_144961	Myosin, heavy polypeptide 2
Myoz2	2.20	3.61	NM_021503	Myozenin 2
GM129	2.11	2.70	NM_001033302	Gene model 129 (NCBI)
Cidec	2.10	2.88	NM_178373	Cell death-inducing DFFA-like effector C
Cfd	2.09	2.31	NM_013459	Complement factor D
Chst1	2.04	2.12	NM_023850	Carbohydrated sulfotransferase 1
Genes down-regulated				
Mup2	0.25	0.26	NM_008647	Major urinary protein 2
Apoa2	0.30	0.30	NM_013474	Apolipoprotein A-2
Mup3	0.35	0.35	NM_001039544	Major urinary protein 3
Serpina1b	0.35	0.34	NM_009244	Serine prepetidase inhibitor, clade A, member 1b
Ttr	0.44	0.43	NM_013697	Transthyretin
Serpina1d	0.46	0.44	NM_009246	Serine petidase inhibitor, clade A, member 1d
Tbx1	0.47	0.39	NM_011532	T-box 1
Mup1	0.47	0.48	NM_031188	Major urinary protein 1
LOC620807	0.48	0.50	NM_001081285	Novel member of the major urinary protein gene family

### Effect of βL-diet on survival

Since the enhancement of metabolic rate found in βL-fed mice has been reported in CR, an exercise model and the genetic and pharmacological model of longevity [22], [23], [24], we checked the survival rate of the βL diet mice used in our behavioral and physiological tests (Fig. S1). They showed a 17% increase in mean survival period compared to the control mice (the βL group: 9.1±0.2 months *vs* the control group: 7.8±0.3 months, p<0.001 by the log rank test), whereas the CR group showed 10% (the CR group: 8.6±0.2 months, p<0.05), suggesting that βL was more effective than CR in increasing survival rate. However, compared with the normal life-span of C57BL/6J mice [25], the life-span of the control group used in our study was shorter, probably due to the effects of stress from experimental manipulations, *i. e.*, checking of body weight, food intake and other tests. Thus, the effects of βL on life-span remains obscure and needs to be measured again with a standard protocol.

## Discussion

Studies have shown that NQO1 gene expression is increased by CR [26], [27] and endogenous NQO1 co-substrates, such as coenzyme-Q (CoQ), are decreased in aged tissue [28]. Here we tried to enhance NQO1 activity in aged mice by feeding them βL, an exogenous co-substrate of NQO1 with a higher potency for the long-lasting redox turnover of NAD [29], [30] and found that this treatment mimics the beneficial effects of CR on molecular, cellular and behavioral functions in aged mice.

How do βL-diets produce a similar effect to that of CR? βL could reduce the net calorie intake by decreasing food uptake or enhancing activity at behavioral level. However, the βL-fed mice showed normal consumption of food and activity as control-diet group (Fig. 1). Despite the normal food uptake and activity, they show higher evaporation of heats which may lead to the decrease of net calorie storage in body (Fig. 2D).

Thus, it is plausible that βL may mimic the mechanisms of CR by increasing NAD+/NADH ratio. While CR leads to an increase of NAD^+^ since a low glucose level resulted from low food uptake facilitates the production of ATP by oxidizing NADH to NAD+ [31], βL could directly facilitate the production of NAD+ through the oxidation of NADH by NQO1 [8](Fig. 1A). The increased NAD^+^ can act as a signaling molecule and activate diverse proteins that play a beneficial role in the control of glucose and lipid metabolism, which is eventually deregulated during the aging process [32].

The increased NAD^+^ by βL may also be associated with the activity of AMPK (Fig. 1B). The administration of βL leads to the phosphorylation of AMPK *in vitro* and *in vivo* by modulating the turnover of NAD^+^/NADH. Rapamycin, a drug that inhibits mTOR signaling is known to increase AMPK phosphorylation [33] and leads to an increase in lifespan of mice [34] but its effect on behavioral functions has not been demonstrated.

Despite that indirect evidence of the effects of the βL-diet to ameliorate aging-related decline, is consistent with and support the NAD^+^/NADH ratio theory of aging, an increase in NAD^+^ was not directly measured in tissues obtained from βL-fed mice. Although the acute intravenous administration of βL(5 mg/kg) resulted in a measurable increase in NAD^+^ molecules *in vivo* (Fig. 1A), the administration of βL in the diet (approximately 70–80 mg/kg/day) may have induced a transient change in the NAD^+^/NADH ratio at the cellular level, which was difficult to maintain during tissue preparation. In addition, the normal NAD^+^/NADH ratio seemed to be tightly linked to a fast metabolic rate *in vivo* (Fig. 1C), *i.e.*, the production and consumption of NAD^+^ are enhanced simultaneously. Further studies will be needed to determine the crucial factor in these experiments: the ratio of NAD^+^/NADH, or their turnover rate with a constant NAD^+^/NADH ratio, or both.

Although the effect of βL on longevity is not fully characterized yet, it significantly enhances body function as measured by motor and cognitive performances of aged mice (Fig. 4), which have been the most critical factors for health span. For example, the age-related loss of muscle function (sarcopenia) directly interferes with behavioral performances and leads to a poor quality of life. Administration of βL leads to enhanced motor function and prevent mitochondrial degeneration, which is a major cause of muscular atrophy [35] without exercise or reduction of food intake that are known to reduce age-related skeletal myopathies [36]. In addition, the enhanced motor functions in βL-fed mice can be explained by prevention of the age-dependent decline of motor functions in the brain [37] since βL also increases the capacity of memory functions and prevents the loss of synapses in aged mice compared with control mice (Fig. 4B, S2).

Finally, these results strongly support the NAD^+^/NADH redox theory of aging that the deregulation of the ratio leads to aging and age-related abnormalities [1], [38], [39], [40] and suggest that the dietary or medical administration of NQO1 co-substrate could be a useful strategy for enhancing the quality of life in aged people who have suffered from muscular and brain dysfunctions. In addition, the content and nutrition of natural co-substrates of NQO1 in food should be re-evaluated in terms of increasing health span.

## Supporting Information

Figure S1 **Kaplan-Meier survival curves.**
(TIF)

Figure S2 **βL increases spine synapse in the brain.** (A) Electron microscopy (×15,000 magnification) of skeletal muscle and brain. Scale bar represents 2 µm. (B) Size of individual mitochondria in hippocampal CA1 region after 10 months treatment (analysis of seven images per mouse, n = 5 per group) (C) Spine synapse number per 100 µm^2^ of hippocampal CA1 region. *P<0.05; **P<0.001 versus control; Data represent the mean ± s.e.m.
(TIF)
