# Supplementary figures and images for: Beta-Lapachone, a Modulator of NAD Metabolism, Prevents Health Declines in Aged Mice
Source: PLoS One. 2012 Oct 11;7(10):e47122. doi: 10.1371/journal.pone.0047122 (PMC3469505; doi:10.1371/journal.pone.0047122)

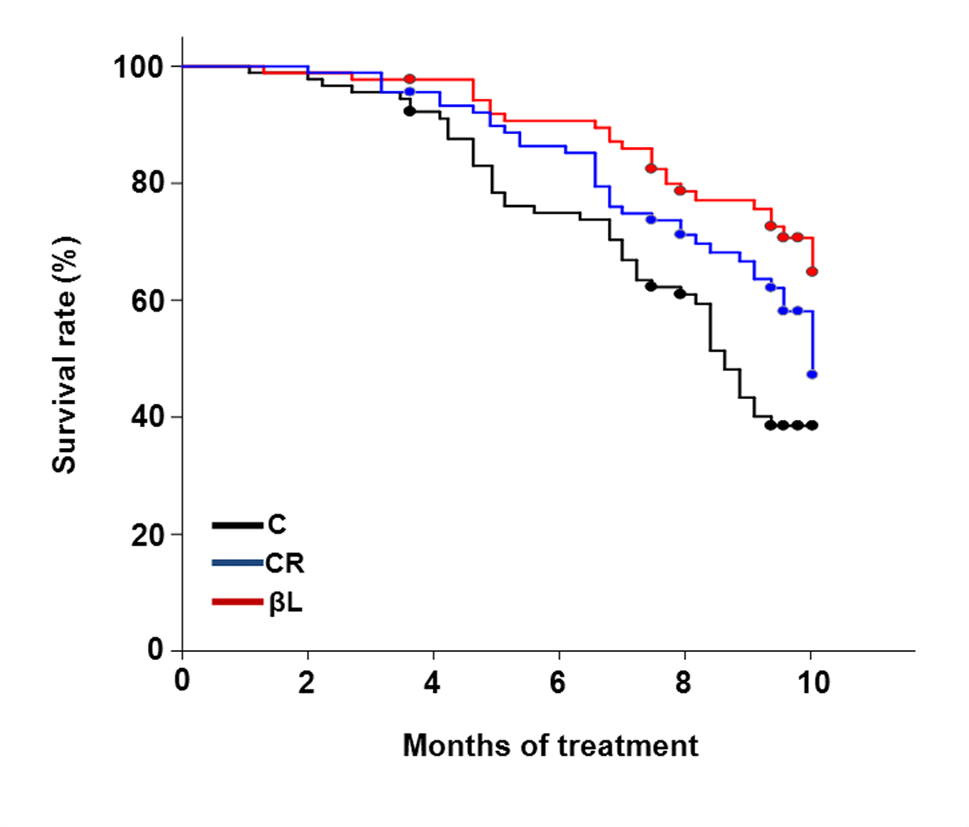

Supplement: Figure S1 — Kaplan-Meier survival curves. (TIF) [file pone.0047122.s001.tif]
